# Proliferation and ovarian hormone signaling are impaired in normal breast tissues from women with *BRCA1* mutations: benefit of a progesterone receptor modulator treatment as a breast cancer preventive strategy in women with inherited *BRCA1* mutations

**DOI:** 10.18632/oncotarget.9638

**Published:** 2016-05-26

**Authors:** Laudine Communal, Myriam Vilasco, Justine Hugon-Rodin, Aurélie Courtin, Najat Mourra, Najiba Lahlou, Morwenna Le Guillou, Muriel Perrault de Jotemps, Marie-Pierre Chauvet, Marc Chaouat, Pascal Pujol, Jean Feunteun, Suzette Delaloge, Patricia Forgez, Anne Gompel

**Affiliations:** ^1^ UMRS 1007, Saints Pères, Paris Descartes University, Paris, France; ^2^ UF de Gynécologie-Endocrinienne, Paris Descartes University, AP-HP, Hôpital Cochin, Paris, France; ^3^ Service d'Anatomie et Cytologie Pathologiques, AP-HP, Hôpital Saint-Antoine, Paris, France; ^4^ Service de Biologie Hormonale, Paris Descartes University, AP-HP, Hôpital Cochin, Paris, France; ^5^ CNRS UMR8200 Gustave Roussy, Stabilité génétique et Oncogénèse, Paris-Saclay University, Villejuif, France; ^6^ Service de Chirurgie Plastique et Reconstructrice, Clinique Hartmann, Neuilly sur Seine, France; ^7^ Département de Sénologie, Centre Oscar Lambret, Lille, France; ^8^ Service de Chirurgie Plastique Reconstructrice et Esthétique et Chirurgie des Brûlés, Denis Diderot University, AP-HP, Hôpital Saint-Louis, Paris; ^9^ Centre Hospitalier Universitaire, Montpellier University, Montpellier, France; ^10^ Breast Cancer Group, Gustave Roussy Cancer Campus, Villejuif, France

**Keywords:** BRCA1, breast cancer, ovarian hormones, prevention, ulipristal acetate

## Abstract

Women with inherited *BRCA1* mutations have an elevated risk (40-80%) for developing breast and ovarian cancers. Reproductive history has been reported to alter this risk, suggesting a relationship between ovarian hormone signaling and *BRCA1*-related tumor development. *BRCA1* interactions with estrogen receptor (ER) and progesterone receptor (PR) signaling were previously described in human breast cancer cell lines and mouse models. However, few studies have examined the effect of ovarian hormone regulation in normal human breast tissues bearing a heterozygous *BRCA1* mutation. This study compares the proliferation level (Ki67) and the expression of ER, PR, and of the PR target gene, fatty acid synthase (FASN), in histologically normal breast tissues from women with *BRCA1* mutations (*BRCA1^+/mut^*, n=23) or without *BRCA1* mutations (*BRCA1^+/+^*, n=28). *BRCA1^+/mut^* tissues showed an increased proliferation and impaired hormone receptor expression with a marked loss of the PR isoform, PR-B. Responses to estradiol and progesterone treatments in *BRCA1^+/mut^* and *BRCA1^+/+^* breast tissues were studied in a mouse xenograft model, and showed that PR and FASN expression were deregulated in *BRCA1^+/mut^* breast tissues. Progesterone added to estradiol treatment increased the proliferation in a subset of *BRCA1^+/mut^* breast tissues. The PR inhibitor, ulipristal acetate (UPA), was able to reverse this aberrant progesterone-induced proliferation. This study suggests that a subset of women with *BRCA1* mutations could be candidates for a UPA treatment as a preventive breast cancer strategy.

## INTRODUCTION

The BRCA1 protein is involved in many essential cellular processes that include DNA damage signaling and repair, cell-cycle control, protein ubiquitination, cell differentiation, and gene transcription regulation, all of which are associated with its tumor suppressor function [1, 2]. Women with heterozygous *BRCA1* mutations have a greater risk of developing breast and ovarian cancer in their lifetime. *BRCA1*-related cancers generally occur in younger women, before the age of menopause, and are more aggressive than breast cancers that arise in the general population [3, 4]. Although sex and organ-specific penetrance of *BRCA1*-related cancers remains poorly understood, ovarian hormones have been implicated in early cell transformation events. Early menarche and late menopause were associated with an increased risk [5, 6]. Prophylactic salpingo-oophorectomy in women with *BRCA1* mutations decreases breast cancer risk by 50% [7, 8], or more if the oophorectomy is performed before the age of 40 [9].

BRCA1 has been shown to play a role in the regulation of estrogen receptor (ER) and progesterone receptor (PR) signaling. Rosen *et al*. demonstrated that BRCA1 inhibited estradiol (E2)-dependent gene transcription [10, 11]. In addition, cross-talk between BRCA1 and ERα was revealed through BRCA1 enhancing transcription of ERα, while ERα in turn increased the transcription of *BRCA1* [12, 13]. Similarly, physical interaction between BRCA1 and PR inhibited PR-dependent gene transcription and increased degradation of PR by the proteasome [14, 15]. Normal breast tissues from women with *BRCA1* mutations did not have different levels of ERα expression compared to non-mutated *BRCA1^+/+^* breast tissues [16]. However the expression of an ER-inducible gene involved in the migration of human breast cancer cells, the trefoil factor 1 (TTF1 or pS2), was decreased in *BRCA1^+/mut^* tissues [16, 17]. A decrease in expression for both isoforms of PR (PR-A and PR-B) was also observed in *BRCA1^+/mut^* tissues, with a ratio in favor of PR-A [16]. In addition, *p53^−/−^*/brca1*^f11/f11^* mice that were treated with progesterone (P4) alone and in combination with E2 had enhanced mammary gland proliferation and developed mammary tumors [18]. Interestingly, these effects were reversed by mifepristone, a PR antagonist. These data, along with studies that report 80% of *BRCA1*-related tumors are negative for ER and PR expression [19, 20], suggest that alterations in hormone signaling contribute to early stages of breast cancer development in histologically normal *BRCA1^mut/+^* cells.

Selective hormone receptor modulators are increasingly considered as preventive breast cancer treatments. Five years of selective ER modulator (SERM) therapy reduced the occurrence of breast cancer in high risk women by 50% [21-25]. Although there are strong implications of PR involvement in *BRCA1*-related breast carcinogenesis, the effect of selective PR modulators (SPRMs) on breast cancer prevention has not yet been evaluated in humans. Among the SPRMs, ulipristal acetate (UPA) was launched as a new generation emergency contraceptive pill and proposed as treatment for uterine fibroids symptoms [26, 27]. Wide use of UPA in the gynecological and clinical fields is due to its ability to efficiently inhibit PR signaling while reducing adverse effects, even with repeated use [28-30].

In this study we analyzed ER and PR expression and responses by immunohistochemistry (IHC) in normal breast tissues from women with heterozygous *BRCA1* mutations (*BRCA1^mut/+^* tissues) or from women without *BRCA1* mutation (*BRCA1^+/+^* tissues). Fresh tissues were also grafted in hormone-treated mice. We report findings that further support the involvement of ovarian hormones in *BRCA1*-related tumor development and support the use of SPRM treatment for breast cancer prevention.

## RESULTS

### Analysis of marker expression in control and BRCA1-mutated breast tissue

Expression of several markers was analyzed by IHC in histologically normal breast tissues from 28 women selected as controls (*BRCA1^+/+^*)** and 22 women with *BRCA1* mutations (*BRCA1^mut/+^*)**. Characteristics of patients bearing *BRCA1* mutations are described in Table 1.

**Table 1:** Clinical features of patients with *BRCA1* mutations

Case	Age	*BRCA1* Mutation	Salpingo-oophorectomy (age at surgery)	Pregnancy & parity
1	56	NA	bilateral (32)	Pr4Pa2
2	39	NA	bilateral (38)	NA
3	51	NA	bilateral (45)	NA
4	26	NA	none	Pr4 Pa2
5	37	NA	none	Pr2 Pa2
6	45	NA	bilateral (44)	Pr2 Pa2
7	43	1135insA (X339)	bilateral (39)	Pr3 Pa2
8	37	NA	none	Pr1 Pa1
9a	42	3627dupA (E1210RfsX9)	bilateral (41)	Pr1 Pa2
9b	43			
10	48	185delAG	Bilateral (46)	Pr4 Pa4
11	28	2012insT (X635)	none	Pr0 Pa0
12	45	4065del4 (N1355-Q1356>K fsX10)	none	Pr0 Pa0
13	28	130t>A (C44S)	none	Pr0 Pa0
14	50	NA	Bilateral (16 and 47)	Pr1 Pa1
15	36	3481del11 (E1161-S1164>GfsX3)	none	Pr2 Pa2
16	33	1599C>T (X494)	none	Pr3 Pa3
17	36	1731C>T (Q538X)	none	NA
18	55	5083del19 (X1670)	none	Pr0 Pa0
19	36	5382insC	none	Pr3 Pa3
20	39	3960C>T (X1281)	none	Pr2 Pa2
21	46	917-918delTT (S267fs)	bilateral (45)	Pr2 Pa2
22	57	2125-2126insA (G709YfsX3)	bilateral (57)	Pr8 Pa8

Patients who tissues were included in the xenograft experiments are highlighted in grey.

Pr: number of pregnancy; Pa: parity; NA: not available.

**Figure 1:**
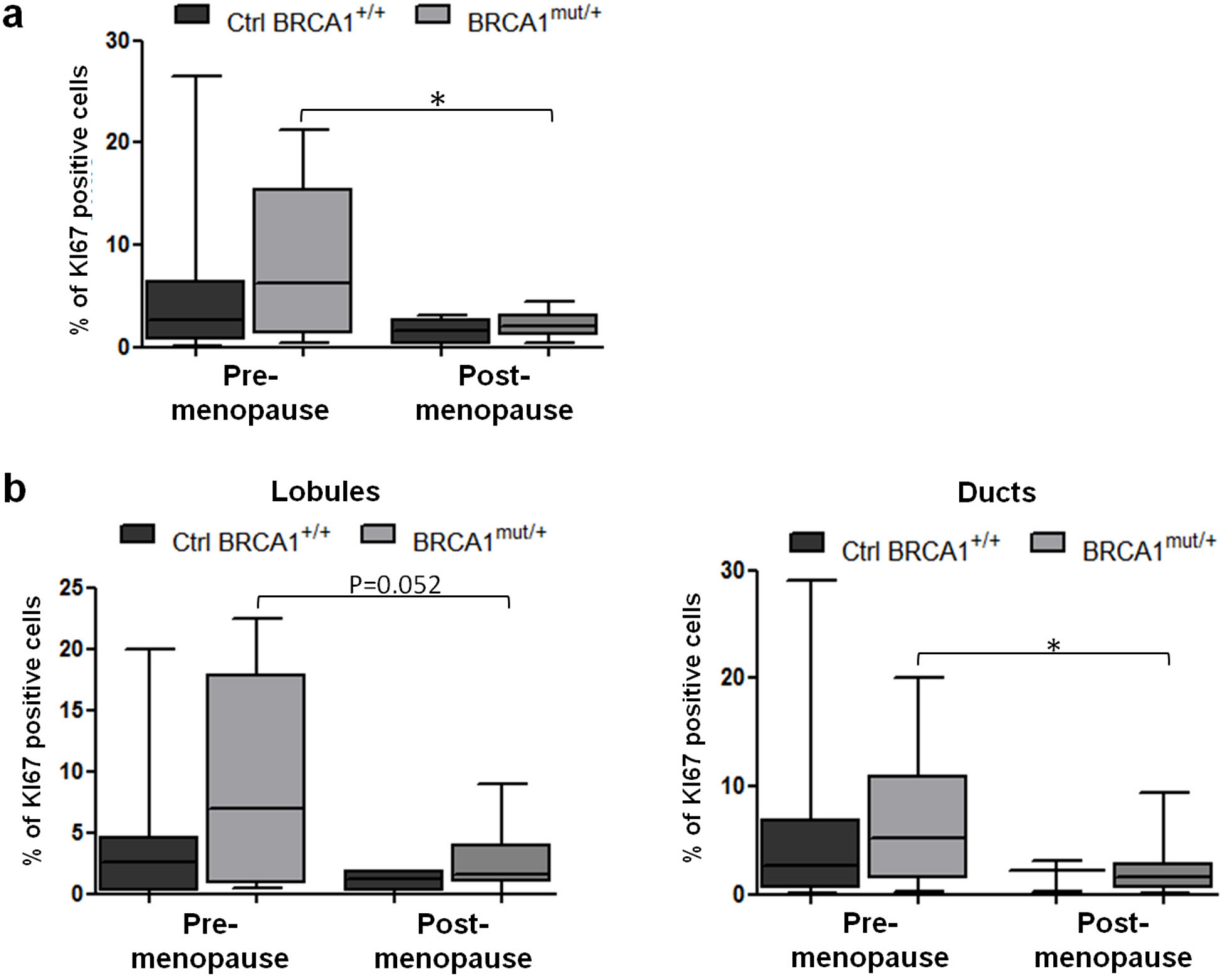
Proliferation status in control and *BRCA1^mut/+^* breast tissues according to menopausal status Tissue sections were stained for Ki67 by IHC. **a.** Quantification of Ki67-positive cells in control (Ctrl *BRCA1^+/+^*) and *BRCA1^mut/+^* breast tissues, pre- and post-menopause. **b.** Quantification of Ki67-positive cells in lobules (left panel) and ducts (right panel) from control and *BRCA1^mut/+^* breast tissues, pre- and post-menopause. Each box contains the interquartile range values with the central line indicating the median value and whiskers extending to the minimum and maximum values. * = p<0.05.

Proliferation marker Ki67 was quantified in luminal epithelial cells of breast tissues. The Ki67-positive cell percentage was similar between the control (4.7 ± 1.3%) and mutated breast tissues (4.6 ± 1.2%) (p=0.974, data not shown). However, when women were sorted according to their menopausal status, we observed that Ki67 expression was significantly reduced in the post-menopause group compared to the pre-menopause group, among patients with *BRCA1* mutations (p=0.019) (Figure 1a). Similar results were observed in lobular and ductal structures of breast tissues when analyzed independently (Figure 1b).

**Figure 2:**
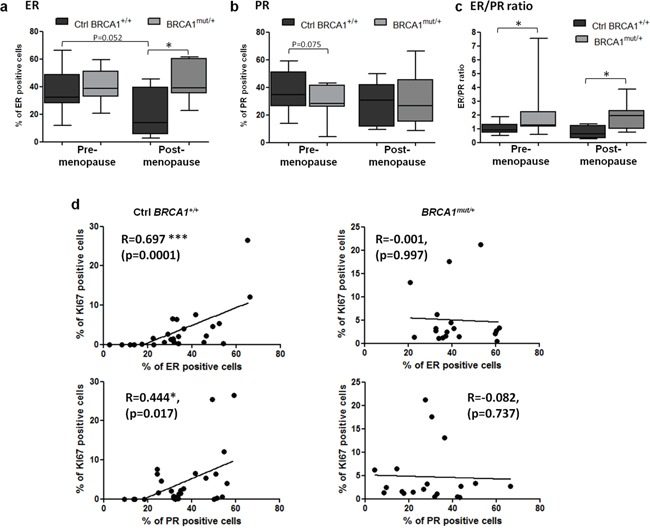
ER and PR expression levels in control and *BRCA1^mut/+^* breast tissues according to menopausal status Tissue sections were stained by IHC for ER or PR as indicated. **a, b.** Percentage of ER and PR positive cells scored in control (Ctrl *BRCA1^+/+^*) and *BRCA1^mut/+^* breast tissues, pre- and post-menopause. **c.** ER to PR percentage ratio was calculated in control and *BRCA1^mut/+^* breast tissues, pre- and post-menopause. Each box contains the interquartile range values with the central line indicating the median value and whiskers extending to the minimum and maximum values. **d.** Correlation curves between Ki67 and hormone receptor expression in control and *BRCA1^mut/+^* breast tissues. Spearman correlation coefficients (R) are indicated. * = p<0.05; ** = p<0.001.

As this result suggested a different sensitivity to gonadal hormones in *BRCA1* mutated tissues compared to control tissues under different ovarian hormonal stimulation, hormone receptor levels were analyzed. Overall, the percentages of ER-positive epithelial cells were not significantly different between *BRCA1^mut/+^* tissues (41.67 ± 2.9%) and control *BRCA1^+/+^* tissues (33.5 ± 3.3%) (p=0.078, data not shown). When analyzed according to menopause status, ER-positive cells were elevated in post-menopausal *BRCA1^mut/+^* tissues in comparison to control tissues (p=0.0162, Figure 2a). A similar profile of expression was observed in lobular and ductal structures (Supplementary Figure 1a).

PR levels were also measured. The percentage of PR-positive cells were not significantly different in *BRCA1^mut/+^* breast tissues (29.52 ± 3.3%) compared to the control group (35.8 ± 2.6%) (p=0.13, data not shown). However, pre-menopausal *BRCA1^mut/+^* breast tissues appeared to have a slightly lower percentage of PR-positive cells compared to control tissues (p=0.075, Figure 2b). We also observed that PR expression was significantly reduced in the lobules from pre-menopausal *BRCA1^mut/+^* group (p=0.042) but not in the ducts (Supplementary Figure 1b). PR levels in lobular structures were reduced after menopause in the control group (p=0.017) but not in the *BRCA1^mut/+^* group (Supplementary Figure 1b).

The ER/PR ratio was calculated for each patient breast tissue. This ratio was significantly elevated in the *BRCA1* mutated group compared to the control group: 2.27 ± 0.90 vs 1.03 ± 0.09 (p=0.029) for pre-menopause and 1.85 ± 0.30 vs 0.73 ± 0.21 (p=0.028) for post-menopause (Figure 2c). Furthermore, strong correlations were observed between Ki67- and ER-positive cells and between Ki67- and PR-positive cells in control tissues (Figure 2d). In contrast, there were no correlation with *BRCA1^mut/+^* breast tissues (Figure 2d), suggesting that the regulation of epithelial breast cell proliferation by hormone receptor pathways is altered in *BRCA1^mut/+^* breast tissues.

**Figure 3:**
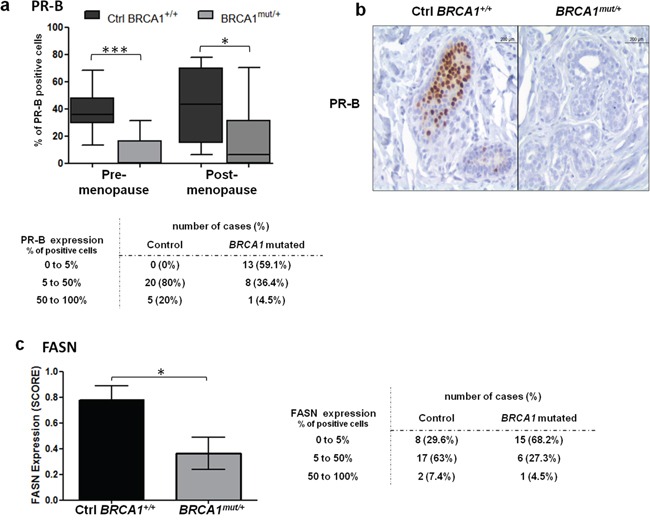
PR-B and FASN expression levels in control and *BRCA1^mut/+^* breast tissues Tissue sections were stained by IHC for PR-B or FASN as indicated. **a.** PR-B positive cells quantified in control (Ctrl *BRCA1^+/+^*) and *BRCA1^mut/+^* breast tissues, pre- and post-menopause. Each box contains the interquartile range values with the central line indicating the median value and whiskers extending to the minimum and maximum values. Table: PR-B positive cells indicated for control and *BRCA1* mutated breast tissues without discrimination of menopausal status. **b.** PR-B stained IHC sections of control and *BRCA1^mut/+^* breast tissues. **c.** FASN expression was scored in control and *BRCA1^mut/+^* breast tissues as described in the Materials and Methods. Table: FASN positive cells indicated for control and *BRCA1* mutated breast tissues without discrimination of menopausal status. * = p<0.05; ** = p<0.001.

The two isoforms of PR, PR-A and PR-B, are responsible for transcriptional activation of distinct and isoform-specific set of genes. Based on previous findings that showed PR-B as the most active isoform for gene transcription, we analyzed its expression [31, 32]. Interestingly, we observed a significant drop in PR-B levels in *BRCA1^mut/+^* tissues compared to control breast tissues, regardless of menopausal status (Figure 3a-3b). In *BRCA1^mut/+^* tissues, 59.1% of samples displayed loss of PR-B expression whereas PR-B was present in all control tissues. PR-B depletion was observed both in lobular and ductal structures from *BRCA1^mut/+^* tissues (Supplementary Figure 1c).

To evaluate the transcriptional activity of PR receptors in *BRCA1* mutated tissues, we examined the expression level of fatty acid synthase (FASN), a PR-induced target gene that is associated with tumor growth of breast cancer cells. FASN catalyzes the synthesis of long chain fatty acids, promoting an altered lipogenic metabolism that is beneficial for cancer cell progression [33, 34]. However, FASN is also involved in the promotion of epithelial differentiation in normal breast cells [35]. FASN mRNA expression is activated by PR in breast tissue and was shown to be specifically induced by PR-B isoform [31, 35-37]. We observed significantly higher FASN levels in control tissues compared to *BRCA1^mut/+^* tissues (p=0.0164, Figure 3c). Sixty eight percent of *BRCA1^mut/+^* samples showed loss of FASN expression whereas only 29.6% of control tissues were negative for FASN expression (Figure 3c).

Altogether our results show that hormone receptor expression was impaired in *BRCA1^+/mut^* tissues compared to control tissues with a marked loss of the PR-B isoform and a decreased expression of the PR target gene, FASN. In addition, proliferation was increased in pre-menopause tissue compared to post-menopause tissue, among women with *BRCA1* mutations. These observations suggest that breast tissues from *BRCA1* mutation carriers have differences in proliferation control and in differentiation driven by hormone receptor levels with reduced levels of PR-B, compared to women without *BRCA1* mutation.

### Response to hormonal treatment in BRCA1 mutated breast tissue xenografts

**Figure 4:**
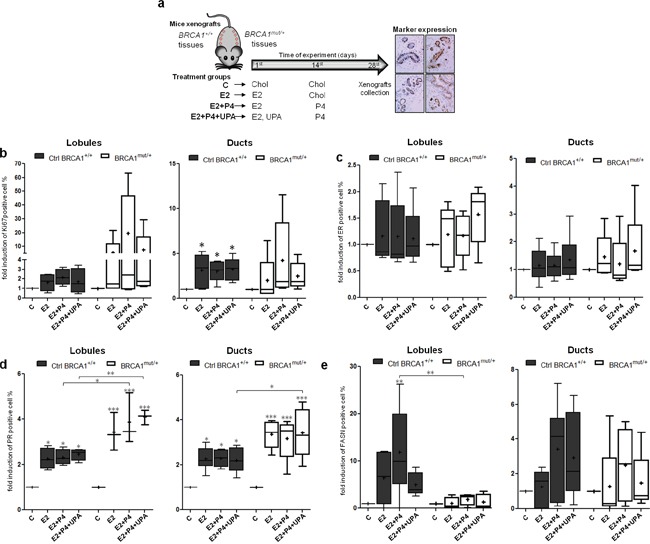
Effects of hormone treatment on *BRCA1^mut/+^* breast tissues xenografted in mice **a.** Illustration of breast tissue xenografts and hormonal treatment strategy. Breast tissue samples from 6 patients without mutations (Ctrl *BRCA1^+/+^*) and from 5 patients with *BRCA1* mutation (*BRCA1^mut/+^*) were xenografted in NMRI^*nu/nu*^ athymic mice. Treatment pellets were grafted in two steps to mimic the menstrual cycle: E2 and UPA pellets were grafted on the first day of the experiment while P4 was grafted on the 14^th^ day. Cholesterol (Chol) was used as a placebo for the control condition (C). After 28 days of treatment, mice were sacrificed and breast tissue xenografts were collected for subsequent scoring of marker expression by IHC. Fold change in induction compared to the control (C) were evaluated for proliferation (Ki67) **b.**, ER **c.**, PR **d.** and FASN **e.** in lobules (left panel) and ducts (right panel) from Ctrl *BRCA1^+/+^* and *BRCA1^mut/+^* breast tissue xenografts. Each box contains the interquartile range values with the central line indicating the median value, the cross indicating the mean value, and whiskers extending to the minimum and maximum values. * = p<0.05; ** = p<0.01; *** = p<0.001.

We studied the cellular responses induced by E2 and P4 in *BRCA1^mut/+^* breast tissues as compared to non-mutated tissues in an NMRI^*nu/nu*^ athymic mouse xenograft model. Four *BRCA1^+/+^* tissue samples and four *BRCA1^mut/+^* tissue samples were grafted subcutaneously onto the backs of mice, on either side of the spine (Figure 4a, see Materials and Methods). Treatments were delivered by pellets inserted under the skin. Time and treatment dose delivery were previously designed to mimic the physiological menstrual cycle in women (Figure 4a and Materials and Methods) [28]. Mice were divided into four groups: Control (C), E2, E2+P4, and E2+P4+UPA.

We analyzed the effects of ovarian hormone treatments on proliferation in lobular and ductal structures (Figure 4b). In *BRCA1^mut/+^* tissues, proliferative responses were highly heterogeneous compared to normal tissues. In *BRCA1^mut/+^* lobules, E2 treatment increased the Ki67 expression from 1 to 21.7 fold, relative to the control. This range of Ki67 expression was drastically increased, from 1 to 63.4 fold, by the addition of P4. Interestingly, the PR inhibitor UPA, reversed the action of P4 and restored a proliferation profile that was similar to mice treated only with E2 (Figure 4b). In ducts, proliferation was significantly increased in the three treatment groups of *BRCA1^+/+^* tissues whereas addition of P4 was the major enhancer of proliferation in *BRCA1^mut/+^* tissues. As observed in lobules, the P4 effect was reversed by UPA (Figure 4b). These results indicate that the proliferative responses to E2 and P4 are deregulated in *BRCA1* mutated breast tissues with a high degree of heterogeneity among patient tissues.

We then quantified ER and PR levels in response to hormonal treatments. ER levels were not modified by hormone treatments in both *BRCA1* mutated and non-mutated tissues (Figure 4c and Supplementary Figure 2). Interestingly PR levels were significantly more elevated by E2 treatment in ductal and lobular structures from *BRCA1* mutated tissues compared to *BRCA1^+/+^* tissues (Figure 4d). As expected, FASN expression was induced by E2+P4 treatment in non-mutated lobules and ducts structures. In contrast, induction of FASN was impaired in *BRCA1^mut/+^* tissues, particularly in lobular structures despite having elevated PR levels (Figure 4e and Supplementary Figure 2). These results suggest alterations in P4 responses and in PR-target gene activation in *BRCA1* mutated tissues.

The heterogeneity of differences were particularly apparent when marker expressions were analyzed independently in *BRCA1^mut/+^* breast tissues from each of the five patients, and compared with the mean response of the six control *BRCA1^+/+^* tissues (Supplementary Figure 2). Responses were homogeneous in the six non-mutated *BRCA1^+/+^* tissues whereas the five *BRCA1^mut/+^* tissues all displayed a different marker expression pattern. Moreover, none of the five *BRCA1^mut/+^* tissues showed the same profile as the *BRCA1^+/+^* tissues. While Ki67 levels in control *BRCA1^+/+^* tissues were only increased by 2.8 ± 0.5 to 3.6 ± 1.3 fold in the presence of E2+P4 relative to the control, Ki67 expression was dramatically elevated in *BRCA1^mut/+^* tissues #17 and #18 (19.5 and 13.0 fold, respectively). The increase in P4-induced proliferation was generally reversed by UPA in these tissues. Interestingly, breast tissues from both patients #17 and #18 were negative for PR-B expression before engraftment (Supplementary Figure 2).

These results highlight the presence of deregulated hormonal responses in *BRCA1^mut/+^* tissues and the heterogeneity of responses among patients. We observed that P4 combined with E2 had an enhanced proliferation effect but dampened PR-dependent gene induction. Interestingly, the use of UPA reversed the P4 effects on proliferation in *BRCA1^mut/+^* tissues.

## DISCUSSION

Hormonal exposure and the overall lifetime number of ovulatory cycles are modifiers of breast cancer risk among *BRCA1* mutation carriers [5, 6, 9]. Here we showed that hormone receptor expression and responses are altered in *BRCA1* mutated breast tissues compared to *BRCA1^+/+^* breast tissues and that these deregulations occur in histologically normal tissues. Since BRCA1 is involved in DNA repair and cell cycle control, our results suggest that E2 and P4 exposure may enhance proliferation in *BRCA1^mut/+^* breast tissues, and potentially increase the accumulation of unrepaired mutations and DNA lesions. Indeed, previous studies have shown that *BRCA1^mut/+^* epithelial breast cells were haploinsufficient for *BRCA1* as they displayed genomic alterations [38, 39], including defects in stalled replication fork repair and a higher frequency in fork collapses [39]. Haploinsufficiency was also involved in impaired differentiation of epithelial luminal cells, leading to an expanded luminal progenitor population in *BRCA1^mut/+^* breast tissues [40]. All these data are consistent with the assumption that *BRCA1^mut/+^* breast cells are likely to cumulate genomic aberrations during mitotic recombination [38]. Higher proliferative rates caused by the hormone signaling in these cells would therefore explain the sex and organ-specific penetrance of *BRCA1*-related cancers.

Currently, prophylactic mastectomy and/or prophylactic annexectomy are used to decrease breast cancer risk in carriers with *BRCA1* mutations; however, there is an urgent need to develop efficient and less aggressive strategies. Here we demonstrated that a PR antagonist inhibited the enhanced proliferative effect of P4 in our *BRCA1^mut/+^* breast tissue xenograft models. PR expression was recently shown to improve the prognosis and treatment response to sporadic ER positive breast cancer by modulating ER functions [41]. However, our results support the use of a PR inhibitor as a potential preventive strategy in women with *BRCA1* mutations, and highlight the effect of *BRCA1* mutations in the regulation of hormone receptors in normal mammary gland. Although mifepristone prevented the onset and development of mammary tumors in *p53^−/−^*/brca1*^f11/f11^* mice [18], this result may only be possible in epithelial cells predominantly expressing the PR-A isoform [42]. In our xenograft experiments, tissues #17 and #18 were associated with the highest P4-induced proliferation levels, and were negative for PR-B before engraftment. The aberrant proliferative effect of P4 was reversed by UPA. Notably, tissue from patient #22 was also negative for PR-B but did not display any hormone-induced proliferation. This may be explained by high levels of breast tissue differentiation [43-45] that the patient most likely experienced through eight full term pregnancies (Table 1). Importantly, UPA did not have any proliferative effect on samples that did not show drastic P4 stimulation. Since E2 also displayed mitogenic action, a combination of an anti-estrogen plus an antiprogestin could be optimal for breast cancer prevention. While tamoxifen has already shown a protective effect against the risk of breast cancer, including contralateral breast cancer, in populations with *BRCA1/2* mutations [21-23, 46], the use of tamoxifen as a standard preventive treatment is limited due to its side effects. A recent study reported higher levels of circulating P4 and E2 in *BRCA1/2* mutation carriers compared to women without *BRCA1/2* mutations [47]. This finding combined with atypical ER and PR profiles in normal breast tissues may also indicate that chemical prevention could be beneficial for these patients. UPA is already used and well tolerated in clinics although further studies are needed to test its potential to decrease cancer risk in women with *BRCA1* mutations.

Deregulated responses to estrogen and progesterone were demonstrated in *BRCA1^mut/+^* breast epithelial cells with a higher proliferation before menopause, suggesting an increased sensitivity to ovarian hormone stimulation. This was further supported by our observations in xenografted tissues where hormone treatments were highly mitogenic in some *BRCA1^mut/+^* tissues compared with non-mutated tissues. In breast cancer cells and mice models, BRCA1 limited ER and PR transcriptional activities and mitogenic actions [10, 15, 18, 48]. Our study supports these findings as the *BRCA1* heterozygous status was associated with an increase of ER and PR proliferative activity. This could explain the reported association between increased hormone exposure and the risk of breast cancer [5, 6, 49]. These results are also supported by the Anderson group study which highlighted the mitogenic effect of E2 in *BRCA1^mut/+^* tissues xenografted in mice [50]. Women with *BRCA1* mutations had an abnormally increased ER/PR ratio that was associated with a striking loss of PR-B receptors. Alternatively, Clarke *et al.* has shown that PR-B expression was lost or decreased in *BRCA1^mut/+^* tissues whereas ER expression was not altered [16]. This is in line with our *BRCA1^mut/+^* data showing a loss of PR-B in almost 60% of samples and little change in ER expression before menopause. Additionally, we observed higher levels of ER in *BRCA1^mut/+^* compared to control tissues from post-menopausal groups, highlighting the importance of the hormonal status in patients with *BRCA1* mutations.

In breast cancer tissue, the typical 1:1 ratio of PR-A and PR-B isoforms in normal epithelial cells is frequently altered due to the apparent loss of PR-B [51, 52]. In T47D breast cancer cells, PR-A overexpression resulted in loss of adherent properties and insufficient FASN mRNA transcription, supporting our observation of decreased FASN protein levels in *BRCA1^mut/+^* tissues [31]. Loss of PR-B expression could be due to increased PR-B degradation or decreased PR-B transcription. Lange *et al*. has shown that ligand-induced transcriptional activity of PR-B was associated with PR-B rapid ubiquitin-dependent degradation, resulting in PR-B loss [53]. However in our study, PR-B was lost without a gain in transcriptional activity as shown by decreased FASN expression. Moreover, BRCA1 is responsible for PR-ubiquitination and its subsequent degradation which is more likely impaired in *BRCA1^mut/+^* tissues, suggesting that another mechanism is responsible for PR-B loss. E2 is the main regulator of transcription of both PR isoforms, and may also contribute to the silencing of PR-B by selective methylation of the promoter under certain conditions [54]. Other possible mechanisms of PR-B loss may include MAPKs which are involved in the control of phosphorylation and turnover of PR-A and PR-B [55]. Alteration of MAPK activities may result in the loss of BRCA1 function [56, 57]. Notably, we showed that *BRCA1* loss of expression was associated with impaired MAPK p38 phosphorylation leading to decreased levels of the activated (S211 phosphorylated) glucocorticoid receptor [56]. Further studies are required to understand the exact mechanisms underlying PR-B loss in *BRCA1^mut/+^* tissues.

The results of our study may also impact the administration of exogenous hormones used for contraception or menopausal hormonal therapy (MHT). The most recent studies reported an elevated risk for breast cancer in women with *BRCA1* mutations if oral contraception was used before the age of 20 years or before the first full term pregnancy [58, 59]. Unlike natural progesterone, contraceptives include synthetic progestins with different affinities for other steroid receptors such as androgen, glucocorticoid or mineralocorticoid receptors, leading to differences in risk for breast cancer. Although further studies are required, the use of MHT after surgical or spontaneous menopause did not appear to increase the risk of subsequent cancers among a small and heterogeneous patient cohort [60-62].

Our study is the first to investigate the effect of *BRCA1* mutations in lobules and ducts separately. Hormone treatment has a greater impact on the terminal ductal lobular unit (TDLU), which is consistent with the observed increase of E2+P4-dependent proliferation in lobules compared to ducts. In women with *BRCA1* mutations, triple negative tumors are predominant but their cellular origin most likely arises from the luminal progenitor [40, 63]. Our results support the idea that these cancers could originate specifically from the TDLU. Analysis of breast tissues according to the menopausal status allowed for differentiation according to hormonal stimulation. However, the number of samples included in our study was low, limiting the strengths of our conclusions. Additional studies are needed to delineate the use of chemoprevention in women with *BRCA1* mutation according to their breast tissues phenotypes.

In conclusion, our findings indicate that *BRCA1* mutation status is associated with alterations in proliferation and in hormone receptor expression and activities in histologically normal breast tissues. These deregulations could participate in the early events of breast cancer development in *BRCA1* mutation carriers. Importantly, for the development of new strategies to prevent the onset of *BRCA1*-related breast cancer, this study suggests that a subset of women with *BRCA1* mutations could be candidates for a UPA treatment as a preventive breast cancer strategy.

## MATERIALS AND METHODS

### Patient recruitment

Normal breast tissues from healthy volunteers were collected between years 2007 and 2012 from various hospital centers in France as part of the BRACAPS consortium cooperation. Breast tissue samples were obtained from women who had signed an informed consent according to the French law on clinical experimentation (L. 1243-3 and L. 1243-4), as part of a biomedical study that included the collection and conservation of cell cultures and xenografts of breast tissues. The authorization number filed for this project is 11826, from the French ethical committee “Comité de Protection des Personnes”.

The cohort included 22 *BRCA1* mutation carriers (*BRCA1*^mut/+^) undergoing prophylactic mastectomies, and 28 women as controls without *BRCA1* mutation (*BRCA1^+/+^*), undergoing breast reductions, and without any reported history of breast disease. The absence of breast malignancy was ensured before and after surgery by breast imaging and anatomopathological review of collected samples, respectively. Hematoxylin–phloxine–saffron staining was used to detect healthy breast tissue.

Women with *BRCA1* mutations had genetic testing that revealed a pathogenic germ-line mutation in the *BRCA1* gene. Among the 22 *BRCA1* mutation carriers, one patient underwent two prophylactic mastectomy surgeries one year apart. For this patient, breast tissues were collected at each surgery and considered as independent samples resulting in n=23 women with *BRCA1* mutation. Clinical characteristics of women bearing a *BRCA1* mutation are described in Table 1.

There was no significant difference between ages at time of surgery for women with or without *BRCA1* mutations. Control women were between 21 to 56 years of age: 37 ± 2.2 years (mean ± SEM). Women with *BRCA1* mutation were between 26 to 57 years of age: 41.6 ± 1.8 years. When hormonal status was uncertain, women above 50 were considered as post-menopausal. Oophorectomized women were included in the post-menopausal group. Premenopausal status was assigned to 23 of 28 women in the control *BRCA1^+/+^* group and to 11 of 22 patients in the *BRCA1* mutation carrier group.

### Mice xenograft experiments

Breast tissue samples were taken from 6 women of the control cohort and from 5 women with *BRCA1* mutations (patients 17, 18, 19, 21 and 22, Table 1) and were xenografted in four week old ovariectomized female NMRI^*nu/nu*^ athymic mice (Janvier laboratory, Le Genest Saint Isle, France). Mean age of the control cohort was 36.0 ± 2.1 years (range: 29-42) and was not significantly different from the mean age of women with *BRCA1* mutation: 46.0 ± 4.5 years (range: 36-57).

Six independent tissue xenograft experiments were conducted as described previously [28]. In four experiments, breast tissues from one control woman and one woman with *BRCA1* mutations were concomitantly xenografted in mice since the dates of patient surgeries were concurrent. One experiment included breast tissue xenografts from two *BRCA1^+/+^* control patients and one other experiment was performed with tissues from only one patient with *BRCA1* mutation. Four tissue fragments per patient were used for subcutaneous xenografts placed on one side of the back of each mouse. Four treatment groups were used per experiment which included the control (C), E2, E2+P4 and E2+P4+UPA (n=4 mice and 16 patient tissue fragments per group). Treatments were delivered by pellets, administered subcutaneously (Figure 4a). Mice were sacrificed 28 days after xenografting, and blood and tissue xenografts were collected. Tissues were immediately fixed in paraformaldehyde solution for IHC analysis. All study protocols and environmental conditions were approved by the French Ethic Charles Darwin committee for the care and use of laboratory animals.

### Immunohistochemistry

IHC analyses were performed using the BOND-MAX workstation (Leica, Nanterre, France) as previously described [28]. Tissue sections were stained with antibodies against Ki67 at 1:100 dilution, ERα at 1:300 (NCL-L-Ki67-MM1 and NCL-L-ER-6F11, Novocastra, Leica, Nanterre, France), PR at 1:80 (MU-328-UC, Biogenex, Fremont, CA, USA) and FASN at 1:400 (sc-20140, Santa Cruz, Dallas, TX, USA). For signal detection, the Bond Polymer Refine Detection kit (Leica) was used. Reagents were purchased from Menarini-Diagnostic (Rungis, France). A negative control (no primary antibody) was included in each set. Marker expression was analyzed as previously described [28].

### Marker analyses

For each marker, the number of positive cells was counted among a total of 1000 lobular and 1000 ductal luminal epithelial cells. The mean percentage of expression was calculated either for all counted cells per section or only for lobular or ductal cells. Breast tissues showing less than 100 lobular and 100 ductal cells were excluded from the analysis. A scoring system was established for FASN quantification of positive cell percentages: 0 (0<5%), 1 (5-50%), 2 (>50%). In xenograft experiments, the final percentage of marker expression was the mean of percentages in tissues from the four mice per treatment group.

### Hormone concentration analyses

Methods and results for measuring serum concentration were described previously [28]. E2 concentration was 36.88 ± 4.25 pg/ml. P4 concentration was 13.05 ± 1.14 ng/ml. UPA concentration was 63.49 ± 10.46 ng/ml which was the same range observed in clinical use [64]. Hormone levels were undetectable in control mice (E2 < 0.8 pg/ml; P4 < 0.4 ng/ml; UPA < 0.5 ng/ml).

### Statistical analysis

Results were expressed as mean ± SEM. Missing values were not considered. The Kolmogorov–Smirnov test and Shapiro–Wilk test were used to test for normality of the group distributions (GraphPad Prism 5, USA). One-way analysis of variance or non-parametric Kruskal-Wallis test followed by Tukey's or Dunn's multiple comparison post-hoc tests were performed according to the normality of the group distributions. When two groups were compared, an unpaired t-test or a non-parametric Mann Whitney test was performed. The Spearman test was used for correlation analysis. A *P*-value < 0.05 was considered significant and n represented the number of independent experiments.

## SUPPLEMENTARY FIGURES


