# Supplementary material for: Proliferation and ovarian hormone signaling are impaired in normal breast tissues from women with BRCA1 mutations: benefit of a progesterone receptor modulator treatment as a breast cancer preventive strategy in women with inherited BRCA1 mutations
Source: Oncotarget. 2016 May 26;7(29):45317–30. doi: 10.18632/oncotarget.9638 (PMC5216725; doi:10.18632/oncotarget.9638)
Supplement: Supplementary file 1 [file oncotarget-07-45317-s001.pdf]

Proliferation and ovarian hormone signaling are impaired in normal breast tissues from women with *BRCA1* mutations: benefit of a progesterone receptor modulator treatment as a breast cancer preventive strategy in women with inherited *BRCA1* mutations

SUPPLEMENTARY FIGURES

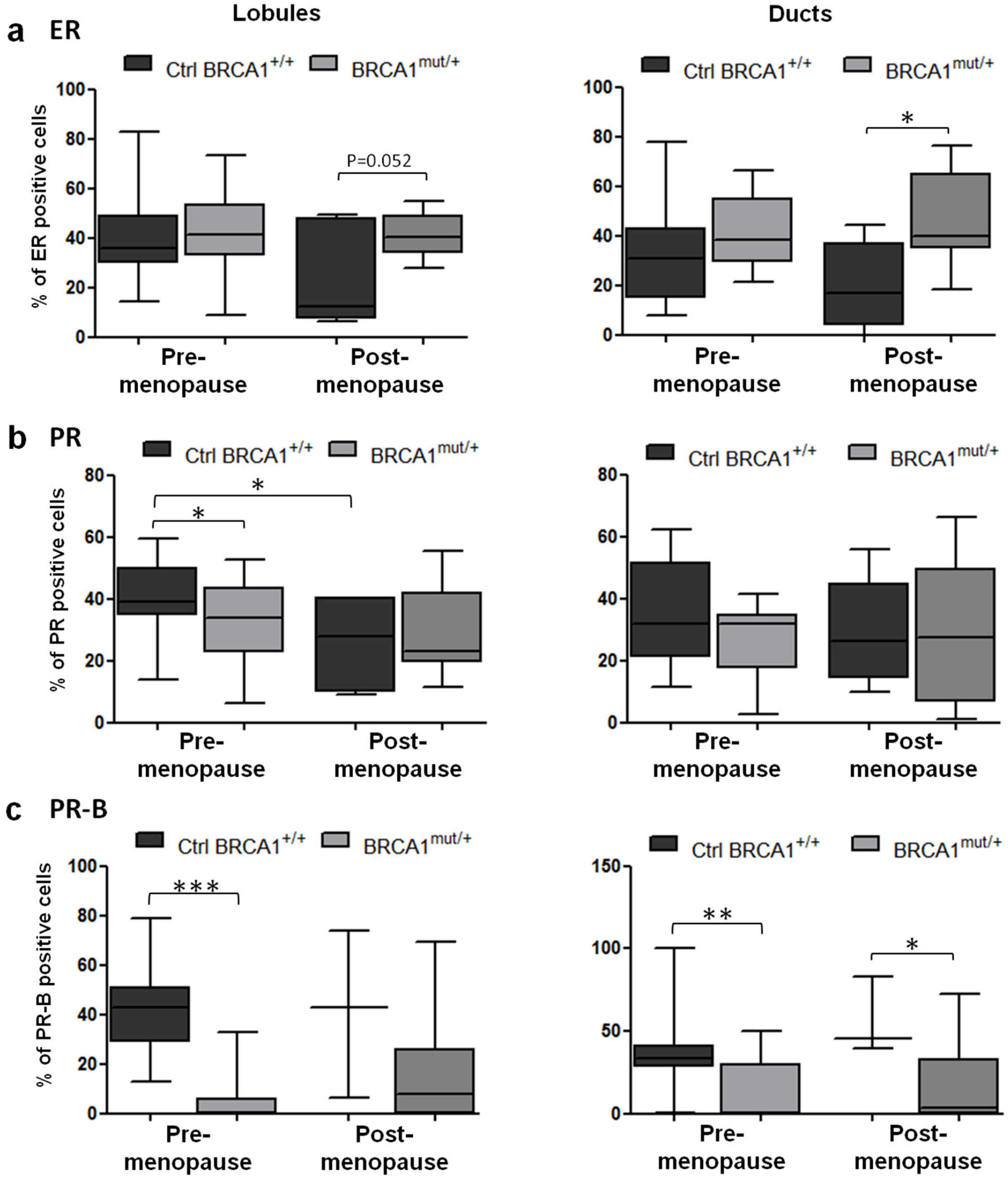

Supplementary Figure S1: Quantification of hormone receptors in lobules and ducts. Quantification of ER **a.**, PR **b.** and PR-B **c.** in lobules (left panel) and ducts (right panel) from control and *BRCA1*$^{mut/+}$ breast tissues, pre- and post-menopause. Tissue slides stained by IHC were scored as described in the Materials and Methods. Each box contains the interquartile range values with the central line indicating the median value and whiskers extending to the minimum and maximum values. * = p<0.05; ** = p<0.01; *** = p<0.001.

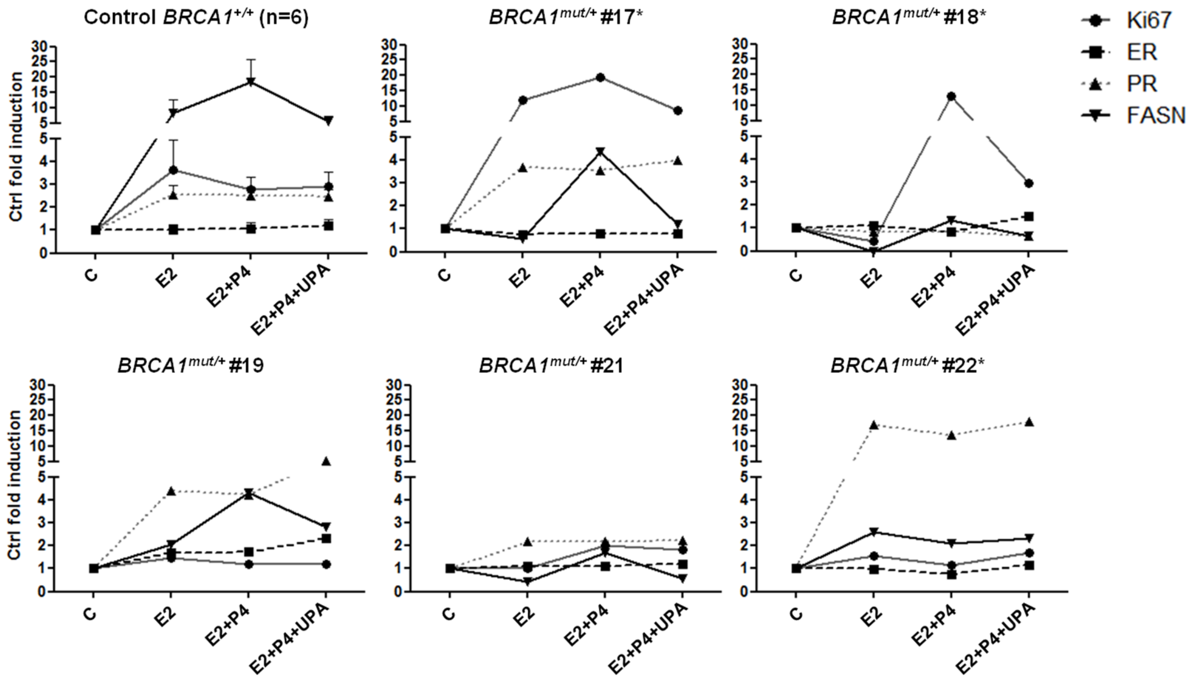

Supplementary Figure S2: Ki67, ER, PR and FASN expression levels in response to hormones in *BRCA1*$^{mut/+}$ patient tissues. a. Profiles of Ki67, ER, PR and FASN induction from hormone treatments as indicated for control *BRCA1*$^{+/+}$ patients (means ± SEM, n=6) and for each woman with *BRCA1* mutations (n=5: #17, 18, 19, 21 and 22). Asterisk (*) indicates patient breast tissues that were negative for PR-B expression before engraftment. Patient #19 and #21 had 12.9% and 36% PR-B positive cells, respectively.
